# Integration analysis of pituitary proteome and transcriptome reveals fertility–related biomarkers in *FecB* mutant Small Tail Han sheep

**DOI:** 10.3389/fendo.2024.1417530

**Published:** 2024-07-23

**Authors:** Xiangyu Wang, Xiaofei Guo, Xiaoyun He, Ran Di, Xiaosheng Zhang, Jinlong Zhang, Mingxing Chu

**Affiliations:** ^1^ State Key Laboratory of Animal Biotech Breeding, Institute of Animal Science, Chinese Academy of Agricultural Sciences, Beijing, China; ^2^ Tianjin Key Laboratory of Animal Molecular Breeding and Biotechnology, Tianjin Engineering Research Center of Animal Healthy Farming, Institute of Animal Science and Veterinary, Tianjin Academy of Agricultural Sciences, Tianjin, China; ^3^ Jilin Province Feed Processing and Ruminant Precision Breeding Cross Regional Cooperation Technology Innovation Center, Jilin Provincial Laboratory of Grassland Farming, State Key Laboratory of Black Soils Conservation and Utilization, Northeast Institute of Geography and Agroecology, Chinese Academy of Sciences, Changchun, China

**Keywords:** FecB, Small Tail Han sheep, fertility, pituitary, transcriptome, proteome, integration analysis

## Abstract

The Booroola fecundity mutation (FecB) in Small Tail Han sheep has been shown to enhance ovulation rates and litter sizes by affecting the hypothalamic–pituitary–gonadal (HPG) axis. Despite the pituitary’s role in reproductive regulation, its involvement in FecB-induced ovulation remains understudied. Our study aimed to fill this gap by analyzing pituitary tissues from FecB homozygous (BB) and wild-type (WW) ewes during luteal and follicular phases using tandem mass tag–based protein quantification and the DIABLO framework for proteomic and transcriptomic data integration. Significant differences in 277 proteins were observed across estrus periods, with network analysis highlighting the voltage-dependent calcium channel L-type alpha-1C as a key convergence point in oxytocin signaling and GnRH secretion pathways. The DIABLO method revealed a strong correlation (0.98) between proteomic and transcriptomic datasets, indicating a coordinated response in FecB ewes. Notably, higher expression levels of Follicle Stimulating Hormone Subunit Beta (FSHB) and Luteinizing Hormone Subunit Beta (LHB) were found in BB ewes during the follicular phase, potentially due to elevated E2 concentrations. Furthermore, our analysis identified genes related to the Gamma–aminobutyric acid type A receptor family (GABRA2, GABRG1, GABRB1) in the pituitary, with GABRB1 showing higher expression in BB ewes. This suggests a role for GABA in modulating GnRH and gonadotropin feedback loops, potentially contributing to the FecB mutation’s effect on ovulation. This study provides novel insights into the pituitary’s role in fertility among FecB sheep, identifying GABA as a potential regulatory factor within the HPG axis. The findings also open avenues for discovering new biomarkers in pituitary endocrinology for sheep breeding purposes.

## 1 Introduction

The Bone morphogenetic mechanism type 1 B (BMPR1B) gene was the first major gene reported to increase ovulation rate, which found in the Booroola prolific sheep strain. BMPR1B^Q249R^ (*FecB*) gene mutation significantly increases the number of ovulations per estrus cycle in ewes (1–3). Recently, Zhou et al. introduced the *FecB* mutation into low–fertility sheep using gene editing technology and observed that this mutation is a crucial causal mutation influencing high sheep productivity (4, 5). Our laboratory results have found that Small Tail Han sheep (STH) carrying the *FecB* mutation exhibit earlier estrus onset and ovulation time compared to wild–type (*WW*) ewes. Additionally, these STHs have smaller mature follicle diameters but significantly higher rates of ovulation and litter sizes compared to their *WW* counterparts (6, 7).

Both *in vivo* pituitary cell and sheep studies at the *in vitro* level showed that follicle stimulating hormone levels were significantly higher in *BB* ewes than in *WW* ewes (*P* < 0.05) (8, 9). In STH sheep, at the 3rd hour after the first estrus, follicle stimulating hormone (FSH) concentrations were significantly higher in *BB* sheep than in *WW* sheep (*P* < 0.05), while luteinising hormone (LH) concentrations were significantly lower than in *WW* sheep (*P* < 0.05) (6). The *FecB* mutation have been observed to potentially lead to a partial attenuation of the transforming growth factor beta (TGFβ) pathway, consequently impacting the follicular response to FSH and LH. Furthermore, this mutation may change the hormonal feedback loop between the follicle and the pituitary gland, thereby influencing the release of FSH and LH (10).

In mammals that ovulate spontaneous like sheep, gonadotropin (FSH and LH) secretion by the pituitary gland drives follicle formation and a massive discharge or surge of gonadotropin that triggers ovulation during a typical ovarian cycle (11). The pituitary, the “master gland of the body”, secretes hormones that are in essence peptides or glycoproteins. Thus, transcriptomics and quantitative proteomics have expanded our understanding of the pituitary endocrine and pituitary regulation of reproduction (12–18). However, these studies have mainly focused on pituitary regulation of estrus, and the molecular mechanisms of pituitary involvement in follicular development to regulate litter size remain poorly understood with the *FecB* mutations. Integration of systems biology and innovative data can unravel complex biology with new insights (19).

In this experiment, we collected pituitary tissues from luteal and follicular phases of STH ewes with different *FecB* genotypes, and detected the changes in protein abundance in these tissues by using tandem mass tagging (TMT) quantitative proteomics and parallel reaction monitoring (PRM) methods. Previously, our group obtained transcriptomic data from these tissues. We performed a combination of these two histological data to screen for core biomarkers, signaling pathways, and biological processes associated with *FecB* gene mutations, in the pituitary gland, that are involved in ovulation.

## 2 Materials and methods

### 2.1 Study design and tissues collection

TaqMan MGB method was used to detect *FecB* mutant genotypes *FecB* (6) in 2–4 years old, similar weight and non–pregnant ewes in the core flock of STH in Yuncheng County, Shandong Province, China. The study was conducted in accordance with the Declaration of Helsinki, and approved by the Animal Ethics Committee of the Institute of Animal Sciences, Chinese Academy of Agriculture Science (No. IAS 2019–49).

**Figure 1:**
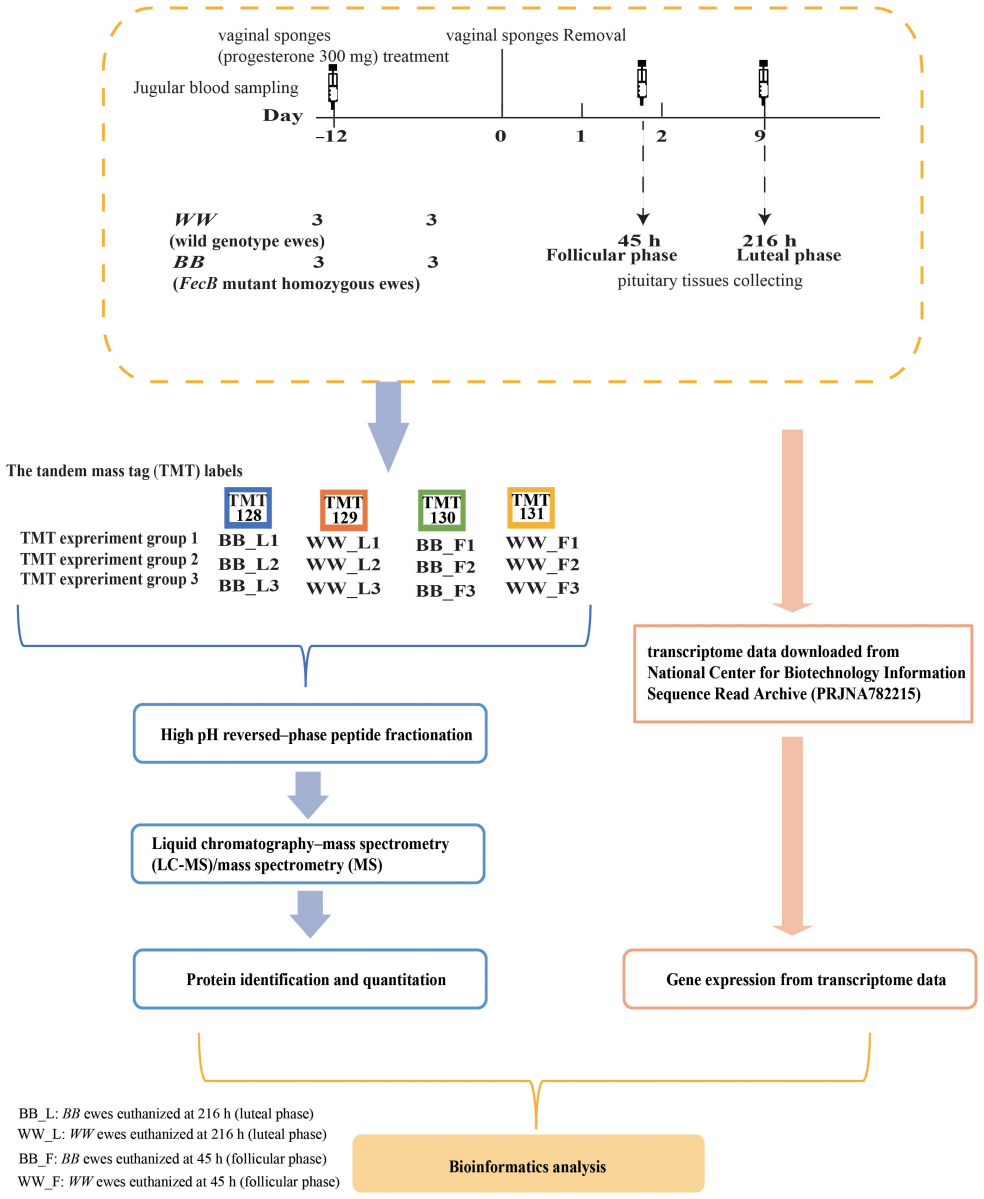
Proteomic and transcriptomic experiment sample group and process of data acquisition and analysis.

Six *WW* genotyped ewes and six *BB* genotyped ewes were then selected based on the *FecB* genotype and reared under natural light conditions with ad libitum feeding. Vaginal sponges (progesterone 300 mg) (InterAg Co., Ltd., New Zealand) were used for 12 days of simultaneous estrus in the above sheep. We set the time of vaginal sponge removal at 0 h (around 10:00 am), and then euthanized 3 *WW* ewes and 3 *BB* ewes at 45 h (follicular phase, F) and 216 h (luteal phase, L), respectively, and obtained pituitary tissues, and then transferred those 12 tissues to –80°C for storage after snap–freezing in liquid nitrogen. Jugular venous blood samples were collected from sheep at the time of vaginal sponge removal (0 hours) and at the time of euthanasia (45 and 216 hours). The concentration of estradiol (E_2_) was measured using an E_2_ RIA kit (BNIBT, China) with a sensitivity of <2 pg/mL, while progesterone (P_4_) levels were quantified using RIA kits (BNIBT, China) with a sensitivity of <0.1 ng/mL. To validate the precision of hormone detection, triplicate assays were conducted for each sample (**Figure 1**). The data for P_4_ and E_2_ were presented as mean ± standard error, and differences in hormone concentrations between genotypes at the same time were assessed using the T-TEST method, with statistical significance defined as *P* ≤ 0.05.

### 2.2 Peptide preparation and TMT labeling

The proteins from the pituitary gland tissues were extracted using the SDT lysis method (20), and protein concentrations were quantified using the Pierce™ BCA Protein Assay Kit (Thermo Fisher Scientific, USA) (21). Each sample was digested with trypsin using the filter aided sample preparation method, with approximately 200 µg of protein per sample (22). The peptide content was quantitated using ultraviolet spectrophotometry (optical density: 280) for subsequent proteomic experiments. Peptides (100 μg per sample) were labeled using the six plex tandem mass tag of TMT Mass Tagging kit (Thermo Fisher Scientific, USA) according to the manufacturer’s instructions. The labeling was performed on *WW* ewes at the luteal phase (*WW*_L), *BB* ewes at the luteal phase (*BB*_L), *BB* ewes at the follicular phase (*BB*_F), and *WW* ewes at the follicular phase (*WW*_F) using TMT–128, TMT–129, TMT–130, and TMT–131, respectively. Each group consisted of three biological replicates (**Figure 1**).

### 2.3 Peptide fractionation and Liquid chromatography–mass spectrometry/mass spectrometry analysis

The labeled peptides from 12 samples were mixed in equal volumes and subsequently lyophilized. A quantity of 100 μg of mixed peptides was dissolved in 300 µL of 0.1% trifluoroacetic acid. The solubilized peptide solution was then transferred to a High pH Reversed–Phase spin column (Thermo Fisher Scientific, USA), which had been pre–equilibrated with 0.1% trifluoroacetic acid and acetonitrile aqueous buffer. By employing gradient elution with high pH acetonitrile solutions of increasing concentration, ten fractions of column–bound peptides were obtained. Each eluted peptide sample was subjected to vacuum drying, followed by reconstitution with 12 μL of 0.1% formic acid, and the concentration were determined by measuring the absorbance at an optical density of 280.

Each sample was subjected to separation using an Easy nLC nanoflow liquid chromatography instrument (Thermo Fisher Scientific, USA). The column was initially equilibrated with 95% 0.1% formic acid in water (buffer A) employing a Thermo Scientific Acclaim PepMap100 (100 μm*2 cm, nanoViper C18, Thermo Fisher Scientific, USA). Subsequently, the samples were introduced into the column and separated utilizing a Thermo scientific EASY column (10 cm, inner diameter 75 μm, resin 3 μm, C18–A2, Thermo Fisher Scientific, USA). A 1–hour liquid gradient strategy was employed, wherein the concentration of buffer B, a 0.1% formic acid acetonitrile aqueous solution (comprising 84% acetonitrile), was gradually increased from 0% to 35% over the initial 50 minutes. Subsequently, the concentration of buffer B reached 100% within the time frame of 50 to 55 minutes. Finally, the concentration of buffer B was maintained at 100% for the remaining 5 minutes. The flow rate utilized was 300 nL/min.

The fractions of samples conducted through chromatography were analyzed using a Q–Exactive mass spectrometer (Thermo Fisher Scientific, USA). Detection was achieved through positive ions, and the acquisition of peptides and peptide fragments was performed by collecting Top20 mass spectrometry (MS) 2 scan fragments subsequent to each full scan. The parent ion scanning range for the full scan encompassed a range of 300 to 1800 m/z, with a resolution of 70000 at 200 m/z. The Automatic Gain Control (AGC) target was set at 1e6, while the Maximum injection time was limited to 50 ms and the Dynamic exclusion time was set to 60.0 s. The MS2 scan activation type employed is higher energy dissociation (HCD), with an isolation window of 2 m/z. The resolution achieved is 17500 at 200 m/z, while the Normalized Collision Energy utilized is 30eV. Additionally, the Underfill ratio is observed to be 0.1%.

### 2.4 The identification and relative quantification of proteomic and transcriptomic data

The MS/MS spectra data were subjected to analysis using Proteome Discoverer v.1.4 (Thermo Fisher Scientific, USA) and MASCOT engine v.2.2 (Matrix Science, UK). The protein reference database was constructed by translating transcriptome data from sheep used in the proteomic analysis (PRJNA782215) (23). The library searching parameters are detailed in **Supplementary Table S1**. Protein abundance differences were calculated after applying a filtering step, where proteins lacking abundance measurements in two or more samples from each group were excluded. The protein abundances were log2–transformed and normalized after filtration, and differences in protein abundance were determined using linear models for microarray data (LIMMA v 3.52.1) package (24). A factorial design was employed for the calculations, incorporating the factors of genotype (two levels: *WW* and *BB*) and estrus (two levels: F and L), with consideration given to interactions between the two factors. Proteins exhibiting significant differences in abundance were selected based on a *P*–value of less than 0.05 and a fold change in abundance comparison between the two groups greater than 1.2 or less than 0.83 (25).

The transcriptome data (PRJNA782215) was aligned and assembled with the sheep reference genome (Oar4.0: GCF_000298735.2) using HISAT2 (v.2.0.5, http://daehwankimlab.github.io/hisat2/) and String Tie (v.1.3.2d, https://ccb.jhu.edu/software/stringtie/). For the analysis of differentially expressed genes (DEGs), read counts were calculated using HTSeq (v0.6.1, https://lira.no-ip.org:8443/doc/python-htseq-doc/html/index.html). Subsequently, DESeq2 (v1.28.1) (26) was employed to normalize the counts and identify genes with differential expression. The model utilized was consistent with the one employed for identifying differentially abundant proteins (DAPs). The criteria for identifying differentially expressed genes included a *P* value less than 0.05, a corrected *P* value of less than 0.05, and a fold change greater than 2.0 between the two groups.

### 2.5 The integration of proteomic and transcriptomic data

We analyzed the repeatability and reliability of the test samples using Pearson’s correlation and Principal component analysis (PCA). The Data Integration Analysis for Biomarker discovery using Latent variable approaches for Omics studies (DIABLO) framework of mixOmics (v.6.20.0) was utilized to integrate transcriptomic and proteomic data in order to identify correlations between the two datasets. The DIABLO core algorithm is an extension of the partial least–squares method (27). Prior to data integration, transcript count data were normalized to log_2_ (counts per million) (logCPM) using the edgeR package (v.3.38.1) (28). Transcripts with a logCPM value below 0 in more than 75% of the sample set were excluded. Similar filtering procedures were applied to the proteomic data during the identification of DAPs. The DIABLO design matrix incorporated estrus stage and genotype factors, and block linkage was 0.1. The determination of the ideal number of components and variables to incorporate in the final model was accomplished through the utilization of the “perf” and “tune.block.splsda” functions, and subsequently validated via 3 × 5–fold cross–validation (times). The model’s performance was evaluated by calculating the balanced error rate and overall misclassification error rate, while the model’s discrimination accuracy was assessed by calculating the area under the curve of the receiver operating characteristic curve (ROC).

### 2.6 Functional annotation and enrichment analysis of biomarkers

To explore the potential role of proteins in the luteal–follicular phase transition and the role of these biomarkers in *FecB* mutations affecting fertility, Gene Ontology (GO) categories (29) and Kyoto Encyclopedia of Genes and Genomes (KEGG) (30) over–representation analyses were ran by clusterProfiler (v4.4.4) (31). The parameter of pAdjustMethod is false discovery rate (FDR). Top enriched KEGG pathways and GO terms were visualized using the two clusterProfiler package functions dotplot and cnetplot.

### 2.7 Quantitative analysis of selected proteins with PRM

To validate the protein abundance obtained by TMT quantification method, the abundance of randomly selected proteins in Pituitary were quantified using PRM analysis (32). The peptides used for PRM were shown in **Supplementary Table S2**. PRM assay was performed using peptides prepared for TMT analysis, approximately 1 μg of peptide from each sample, mixed with 20 fmol of the standard peptide (SAAGAFGPELSR, Thermo Fisher Scientific, USA). Reversed–phase chromatographic analysis was performed with the Easy nLC–1200 system (Thermo Fisher Scientific, USA) For the chromatographic analysis, the C18 Trap Column was 100 μm * 50 mm, with a resin of 5 μm, and the C18 Analytical Column was 75 μm * 200 mm, with a resin of 3 μm. The chromatographic separation exhibited a flow rate of 300 nl/min, with a gradient characterized by the following changes in concentration: buffer B experienced an increase from 5% to 10% within the initial 2 minutes, followed by a subsequent increase from 10% to 30% between 2 minutes and 45 minutes. From 45 minutes to 55 minutes, there was a further increase from 30% to 100%, and during the final 5 minutes, the concentration of buffer B remained constant at 100%. The fractions of samples conducted through chromatography were analyzed using a Q–Exactive mass spectrometer (Thermo Fisher Scientific, USA). Detection was achieved through positive ions, and the acquisition of peptides and peptide fragments was performed by collecting Top20 MS2 scan fragments subsequent to each full scan. The parent ion scanning range for the full scan encompassed a range of 300 to 1800 m/z, with a resolution of 60000 at 200 m/z. The AGC target was set at 3e6, while the Maximum injection time was limited to 200 ms. The MS2 scan activation type employed is higher energy dissociation (HCD), with an isolation window of 1.6Th. The resolution achieved is 30000 at 200 m/z, while the Normalized Collision Energy utilized is 27eV. The AGC target was set at 3e6, while the Maximum injection time was limited to 120 ms (33). The peptide were extracted and calculated by Skyline v.3.5.0 (34)

## 3 Results

### 3.1 Overview of pituitary proteomic data

Twelve pituitary samples were randomly divided into three experimental groups, with four samples in each group. Care was taken to ensure that each group included one sample from *BB*_F, *BB*_L, *WW*_F, and *WW*_L, thus rendering them appropriate for the six–plex TMT labeling strategy. The triple TMT experiments yielded 4896, 4860, and 5176 peptides, respectively. The subsequent integration of these three sets of results and their annotation ultimately led to the identification of 5836 distinct proteins, as depicted in **Supplementary Figure S1A**, for subsequent analysis. The analysis of protein molecular weight distribution showed that the molecular weights of 99% of proteins were between 6 and 312 kD (**Supplementary Figure S1B**). Approximately, the protein sequence coverage of 1545 proteins were > 30% (**Supplementary Figure S1C**). More than 1541 proteins included less than two unique peptides. The proteins XP_004018043.1, XP_014949338.1, XP_012026869.1, and XP_014952969.1 were identified to have over 100 unique peptides (**Supplementary Table S3**).

### 3.2 Results of PRM quantification

**Figure 2:**
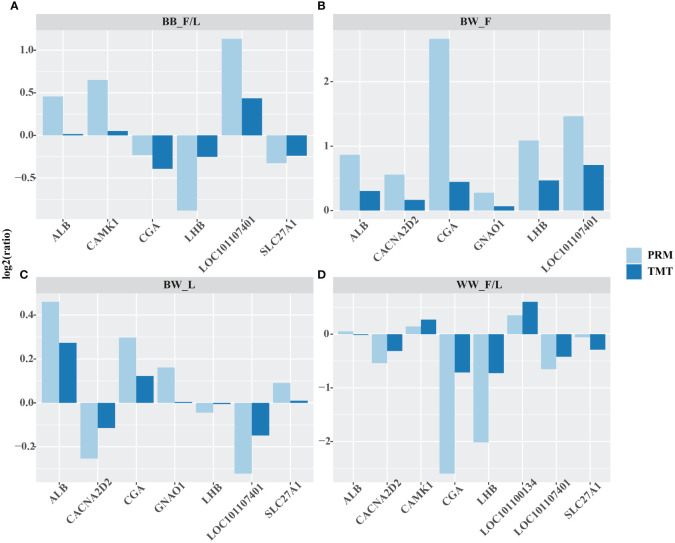
Quantitative validation of the abundance of important proteins obtained by tandem mass tag (TMT) by parallel reaction monitoring (PRM). albumin (ALB), calcium voltage–gated channel auxiliary subunit alpha 2 delta 2 (CACNA2D2), calcium/calmodulin dependent protein kinase I (CAMK1), glycoprotein hormones, alpha polypeptide (CGA), G protein subunit alpha o 1 (GNAO1), luteinizing hormone beta polypeptide (LHB), glutathione S–transferase Mu 1 (LOC101107401), solute carrier family 27 member 1 (SLC27A1), and alcohol dehydrogenase class–2 isozyme 2–like (LOC101110134) were selected for validation of the differences in protein abundance using the PRM in the following subgroups: follicular versus luteal phase in *BB* genotype ewes (*BB*_F/L) group **(A)**, follicular phase in *BB* ewes compared to *WW* ewes (BW_F) group **(B)**, and luteal phase *BB* ewes compared with *WW* ewes (BW_L) groups **(C)**, and follicular versus luteal phase in *WW* genotype ewes (*WW*_F/L) group **(D)**. The log2(ratio) values were computed between the aforementioned groups. The protein abundance, as determined by the PRM method, exhibited similar trends to that of the TMT method for the selected proteins across all four groups.

Albumin (ALB), calcium voltage–gated channel auxiliary subunit alpha 2 delta 2 (CACNA2D2), calcium/calmodulin dependent protein kinase I (CAMK1), glycoprotein hormones, alpha polypeptide (CGA), G protein subunit alpha o 1 (GNAO1), luteinizing hormone beta polypeptide (LHB), glutathione S–transferase Mu 1 (LOC101107401), solute carrier family 27 member 1 (SLC27A1), and alcohol dehydrogenase class–2 isozyme 2–like (LOC101110134) proteins were selected for validation of the differences in protein abundance using the PRM in the following subgroups: follicular versus luteal phase in *BB* genotype ewes (*BB*_F/L) group, follicular phase in *BB* ewes compared to *WW* ewes (BW_F) group, and luteal phase *BB* ewes compared with *WW* ewes (BW_L) groups, and follicular versus luteal phase in *WW* genotype ewes (*WW*_F/L) group. The differences in protein abundance obtained from TMT versus PRM in the above groups were then expressed as log2 (ratio) values. Comparison of these two methods showed a consistent trend in the differences in protein abundance between the different groups they detected (**Figure 2**, **Supplementary Tables S2, S3**).

### 3.3 The profile of the DAPs in proteomics data

**Figure 3:**
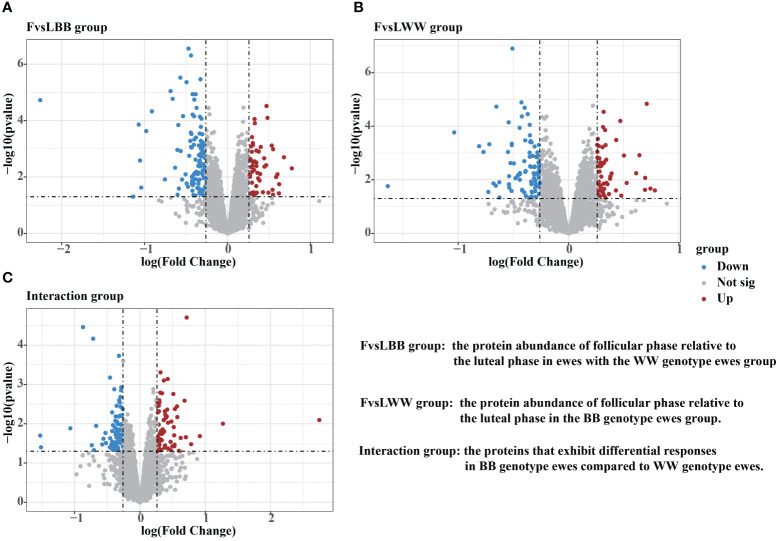
Volcano plots were demonstrating the differentially abundant proteins (DAPs) in FvsLBB group **(A)**, FvsLWW group **(B)** and Interaction group **(C)**. The threshold for determining upregulated and downregulated DAPs was set at a foldchange higher than 1.2 or lower than 0.83, with a P value lower than 0.05.

The effects of genotype and estrus, as well as the interactions between them, were evaluated using the limFit and eBayes functions of the LIMMA package. Then, the contrast function was used to further analyze which proteins changed during the transition from the luteal to follicular phase in *WW*genotype (FvsL*WW*) and *BB* genotype (FvsL*BB*) ewes, respectively. Additionally, the contrast function was used to determine which proteins responded differently (interactions) in *BB* vs. *WW* Following the thresholds described in the Material Methods, 170 DAPs were detected in the FvsL*BB* group. Out of these, 55 proteins were up–regulated during the follicular phase, while 115 proteins were down–regulated (**Figure 3A**, **Supplementary Table S4**). In the FvsL*WW* group during the follicular phase, 144 DAPs were screened, with 54 being up–regulated and 90 being down–regulated (**Figure 3B**, **Supplementary Table S5**). In the interaction group, 145 DAPs were detected, with 73 being up–regulated and 72 being down–regulated for *BB* genotypes relative to the wild type (**Figure 3C**, **Supplementary Table S6**).

**Figure 4:**
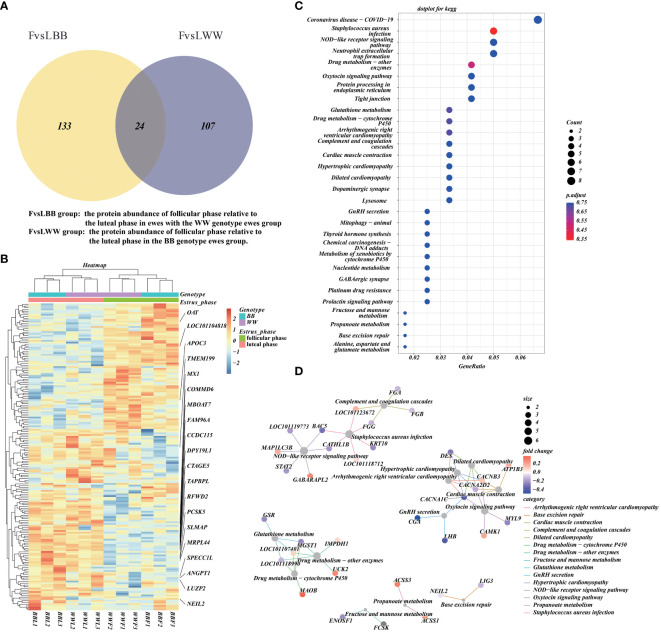
The functional annotation and pathway enrichment of differentially abundant proteins (DAPs) during the luteal–follicular phase transition. **(A)** The Venn plot that illustrates the overlap of DAPs between the FvsL*WW* and FvsL*BB* groups. **(B)** Additionally, a hierarchical cluster analysis is performed on the DAPs in the FvsL*WW* and FvsL*BB* groups, with all protein abundance values normalized and scaled. **(C)** Furthermore, a dotplot is presented, showcasing the top 30 KEGG enrichment terms for the concatenated DAPs in the FvsL*WW* and FvsL*BB* groups. The pAdjust parameter used for statistical analysis is the false discovery rate (FDR). **(D)** Finally, the networks corresponding to the top 30 KEGG enrichment terms are visualized using cnetplot.

We obtained the intersection of DAPs between the FvsL*WW* and FvsL*BB* groups and plotted the Venn diagram (35). The intersection of the FvsL*WW* and FvsL*BB* groups had 37 DAPs (**Figure 4A**). The abundance of a total of 277 unique DAPs in these two groups was then analyzed by hierarchical clustering heatmap, and the 37 DAPs in both groups showed the same trend (**Figure 4B**). We annotated the unique DAPs in these two groups with GO and KEGG enrichment (**Figure 4C**, **Supplementary Tables S7**, **S8**). Network clustering of the top 30 KEGG–enriched terms using cnetplot (**Figure 4D**). Pathways of the Oxytocin signaling pathway and GnRH secretion were linked together with CACNA1C, and this linkage contained 7 proteins, including LHB. The cnetplot also describes the corresponding network of three metabolically relevant KEGG–enriched terms (Glutathione metabolism, Fructose and mannose metabolism, and Propanoate metabolism) (**Figure 4D**), which are related to pituitary endocrine function.

### 3.4 DIABLO multi–omics analysis to screen potential biomarkers associated with reproduction in STH sheep

**Figure 5:**
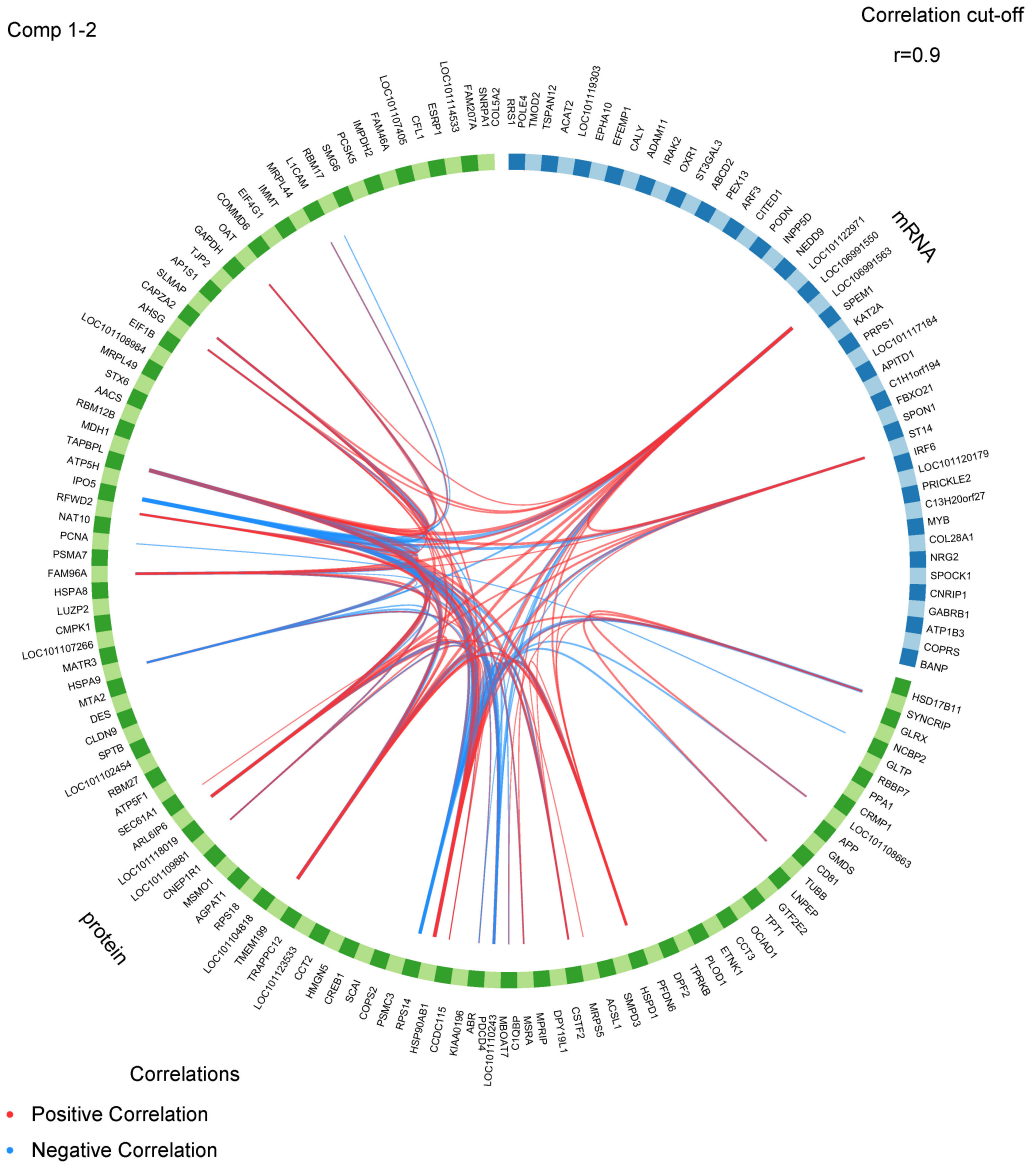
A circos plot displays the positive (red lines) and negative (blue lines) correlations (r > 0.9) between features variables in the quadrants.

The correlation coefficient of the gene expression levels between samples exceeded 0.85 in one group, suggesting a high degree of consistency and reliability in the sample selection (**Supplementary Figure S3**). Proteomic and transcriptomic data from the same ewes were integrated using the DIABLO framework. This supervised learning approach was used to screen for the best classification feature variables from multifactorial data containing follicular stage and genotype. In the follicular phase, there was significant variation in mRNA expression and protein abundance patterns between the two genotypes of type *BB* and *WW* sheep. However, during the luteal phase, the data patterns of the two omcis of type *BB* and *WW* sheep were found to be similar. This observation is illustrated in **Supplementary Figure S2A**. Furthermore, the proteomic and transcriptomic datasets exhibited a strong positive correlation, as evidenced by a Pearson’s correlation score of 0.98 on two components, as shown in **Supplementary Figure S2B**. Furthermore, the scatter plots (**Supplementary Figure S2B**) show that the biomarkers selected by integrating the two omics data can effectively discriminate between data from different estrus stages and different genotypes in the follicular phase. These results suggest that the *FecB* mutation primarily affects ovulation during the follicular phase. The “centroids.dist,” “mahalanobis.dist,” and “max.dist” are used to estimate the classification error rate. The findings derived from a 3 × 5–fold cross–validation demonstrate that the sPLS–DA model exhibits superior performance when ncomp = 3 (**Supplementary Figure S2C**). The circos plot (**Figure 5**) was employed to visualize the positive or negative correlation (**Supplementary Table S9**) between protein abundance and mRNA expression, utilizing ncomp = 1–2 and a correlation cutoff of 0.9. Among these protein–mRNA interaction pairs, 24 exhibited a positive correlation while 10 displayed a negative correlation. These proteins and mRNAs exhibited significant interactions, with three specific mRNAs (COL28A1, LOC106991550, and ST14) demonstrating connectivity. Notably, the LOC106991550 mRNA displayed interactions with a noteworthy total of 20 proteins.

**Figure 6:**
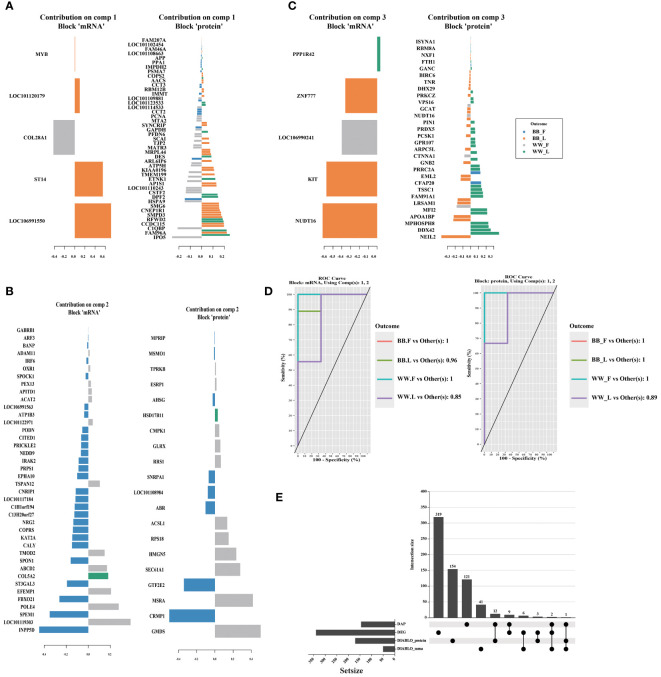
The figure showcases the featured biomarkers and the accuracy of models in proteomic and transcriptomic data through the utilization of DIABLO. The loading vector weights of the prominent biomarkers in different omics are presented individually for component 1–3 **(A–C)**. **(D)** Additionally, the Receiver operating characteristic area under the curve result higher than 0.85 demonstrates the high effectiveness of the DIABLO multi–omics joint analysis in identifying characteristic biomarkers. **(E)** Furthermore, the intersection between differentially abundant proteins (DAPs), differentially expressed genes (DEGs), and the featured biomarkers identified by the DIABLO method is illustrated using the upset plot. *BB*_F, *BB* genotype ewes at the follicular phase; *BB*_L, *BB* genotype ewes at the luteal phase; *WW*_F, *WW* genotype ewes at the follicular phase; *WW*_L, *WW* genotype ewes at the luteal phase.

The plotLoadings function in the DIABLO package was used to visualize the degree of contribution of the featured variables in the three components obtained from the combined analysis. Among the variables in component 1 (**Figure 6A**), 90 were from proteomic data and 5 from transcriptomic data; among the variables in component 2 (**Figure 6B**), 20 were from proteomic data and 40 from transcriptomic data; and among the variables in component 3 (**Figure 6C**), 60 were from proteomic data and 5 from transcriptomic data (**Supplementary Table S10**). The auroc() function is utilized to calculate the Receiver Operating Characteristic (ROC) curve and Area Under the Curve (AUC) for comparing one group against the others. The AUC values, exceeding 0.8, obtained from two blocks of 2 components indicate that the sPLS–DA analysis conducted in this study is effective (**Figure 6D**). The 319 differential expressed genes (DEGs) (fold change (FC) > 2, pvalue < 0.05, **Supplementary Table S11**) and 121 DAPs obtained interaction group were then compared with the featured mRNAs (DIABLO_mRNA) and proteins (DIABLO_protein) selected by DIABLO (**Supplementary Table S10**). The four featured datasets obtained by these two methods differed significantly and did not intersect with each other. CALY and SPON1 were the three datasets intersected by DAP, DEG, and DIABLO_mrna. NUDT16, a DAP, also appeared in the DIABLO_mRNA and DIABLO_protein datasets, respectively. These results were visualized by the online tool ChiPlot (https://www.chiplot.online/) in **Figure 6E**.

**Figure 7:**
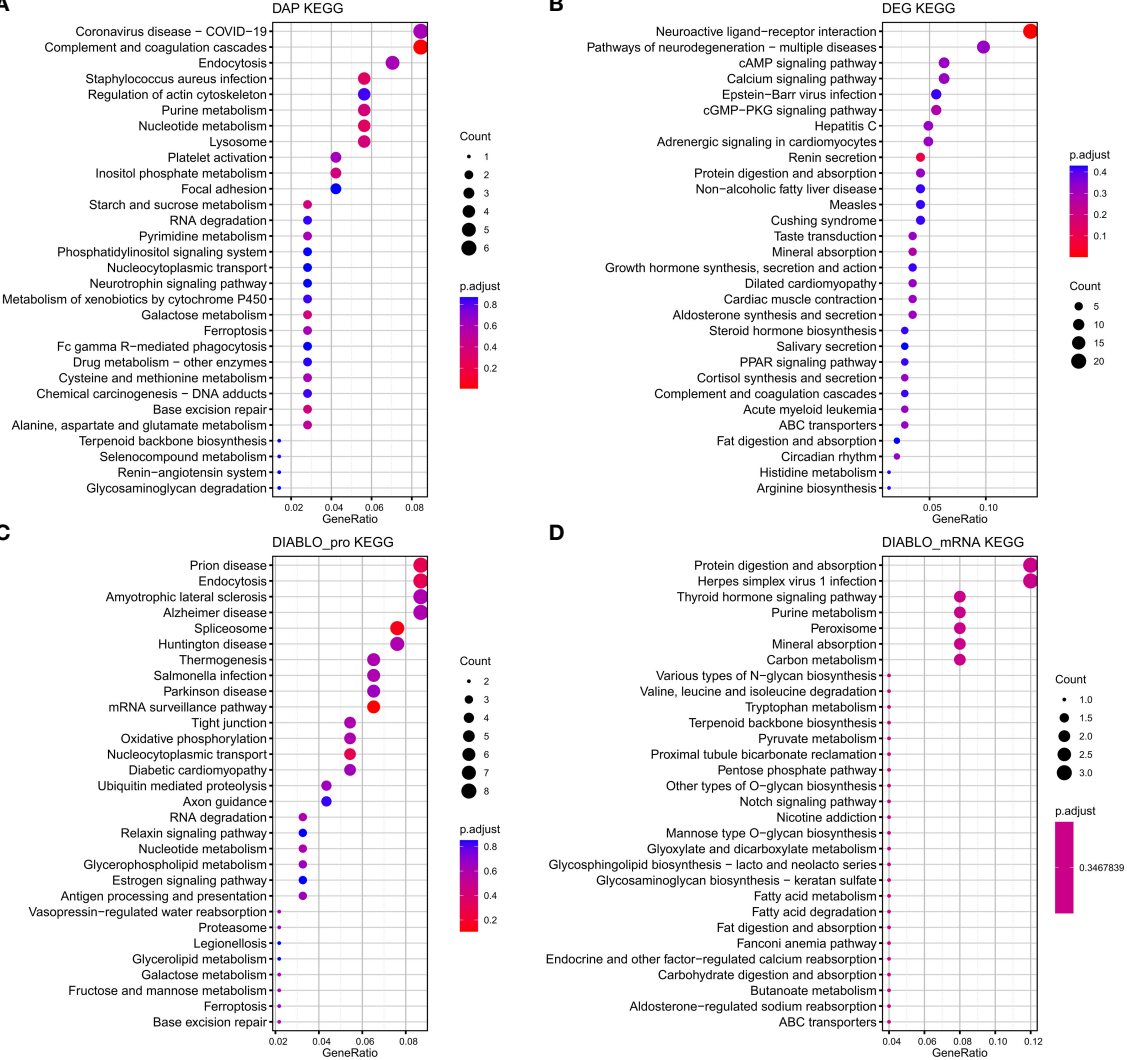
The top 30 KEGG pathways that exhibit enrichment in terms of differentially abundant proteins (DAPs) **(A)** and differentially expressed genes (DEGs) **(B)** within the interaction group, as well as the top 30 KEGG pathways enriched based on the biomarkers selected by DIABLO of protein (DIABLO_protein) **(C)** and mRNA (DIABLO_mRNA) **(D)** block.

The proteins and mRNAs from the four datasets were annotated according to KEGG enrichment analysis (**Supplementary Tables S12–15**). The top 30 KEGG pathways are presented in **Figure 7**. It is interesting to note that some pathways were related to energy and amino acid metabolic pathways related to reproduction processes, such as Cortisol synthesis and secretion, Arginine biosynthesis, Fructose and mannose metabolism, Pyruvate metabolism, and Tryptophan metabolism. These genes are also enriched for pituitary signaling molecules and interactions and related pathways of signal transduction, including the calcium signaling pathway and the cAMP signaling pathway. There were also several hormone–related pathways such as Growth hormone synthesis, secretion and action, Steroid hormone biosynthesis, and estrogen signaling pathway.

## 4 Discussion

The pituitary is an important regulator involved in the hypothalamic–pituitary–gonad axis (HPG) axis and it orchestrate the complex neuroendocrine regulation of reproduction (36). *FecB* mutation may be involved in hormonal regulation and signal transduction during follicular development and ovulation (37). The combined analysis of proteome and transcriptome data provides an effective method to explain complex biological processes and screen valuable biomarkers for breeding (38, 39). Due to RNA–mediated transcription regulation and protein post–translational modification, the correlation between proteome and transcriptome data calculated by using Pearson correlation alone is very low, and it is difficult to effectively use the information of the two omics at the same time (40, 41). We choose DIABLO method, which mainly uses the principle of machine learning to solve the problem of multi–omics data integration mining with small sample size (42). Using this method to reduce the complexity of the two platforms of pituitary transcriptome and proteome data, we calculated the correlation of the two omics to be 0.98, and also screened important signature pathways.

The pituitary gland is an important gland for controlling the physiologic functions of reproduction, and it consists of the adenohypophysis and the neurohypophysis (43). In our proteomic results, we identified reproduction–related hormones secreted by the pituitary, FSH, LH, growth hormone, prolactin and thyroid–stimulating hormone (TSH). We also identified oxytocin synthesized by the hypothalamus and stored in the posterior pituitary (44). The process of follicular growth and ovulation is intricately regulated by interactions between the hypothalamus, pituitary gland, ovary, and uterus (37). The hypothalamus secretes gonadotropin-releasing hormone (GnRH) to stimulate the synthesis and release of FSH and LH from the pituitary gland (45). These hormones then act on the cells of the follicle to support its growth and maturation. As the follicle develops, the granulosa cells utilize androstenedione produced by the endometrium to synthesize E_2_, which in turn inhibits the synthesis and release of FSH and LH from the pituitary gland through negative feedback regulation (46). During the follicular phase, there is a shift in the regulation of GnRH/LH by E2 from negative to positive feedback as the follicle matures, leading to the LH peak prior to ovulation (47). Our analysis revealed that E2 concentrations were significantly elevated in the BB genotype compared to the WW genotype during the follicular phase (**Supplementary Figure S4**), potentially accounting for the increased expression levels of Luteinizing Hormone Subunit Beta (LHB) in the BB genotype. In follicular, via TGFβ/BMP signaling pathway, mutant *FecB* gene carrier produced small antral follicles with a reduced number of granulosa cells exhibiting higher sensitive to FSH, leading to follicular maturation in advance as attested by precocious LH receptor expression (48).

Pituitary, as the intermediary organ of the reproductive axis, also regulate follicle–luteal transition by the synergy between endocrine and local auto/paracrine factors (49). The transition from follicle to corpus luteum was essential to the reproductive cycle and establish pregnancy by producing progesterone (50). During the luteal–follicular phase transition, our Network clustering analysis of the DAP revealed that Voltage-dependent calcium channel L type alpha-1C (CACNA1C) serves as a point of convergence between the Oxytocin signaling pathway and the GnRH secretion pathway (**Figure 4**). The CACNA1C gene, recognized for its role in calcium channel activity, has been pinpointed as a significant candidate gene for the initiation of puberty in Jining grey goats (51). Furthermore, evidence suggests a strong correlation between CACNA1C and GnRH, which collaborate to facilitate ERK activation and subsequent elevation in FSH and LH secretion (52). These observations lead us to propose that CACNA1C plays a pivotal role in the transition from the luteal to the follicular phase. The Oxytocin signaling pathway exhibits enrichment of calcium/calmodulin-dependent protein kinase I (CAMKI), as well as the CAMKI and CACNA1C genes, which are enriched in the calcium signaling pathway. Intracellular Ca^2+^ concentration serves as a crucial signaling molecule in the regulation of exocytosis, controlling the release of neurotransmitters and endocrine hormones (53). The release of pituitary gonadotropins is stimulated by GnRH from the hypothalamus through a Ca^2+^ dependent mechanism (54). Research has demonstrated that the activation of CaMKI by GnRH is essential for the derepression of the FSHB gene through the phosphorylation of various class IIa HDACs. Conversely, the derepression of the LHB-subunit gene does not rely on CaMKI activation (55).

After annotation and enrichment of DEGs of interaction group. we identified the Neuroactive ligand–receptor interaction pathway. This pathway was also enriched in our previous multi–omics analysis of the hypothalamic in different genotypes of *FecB* mutation (56). This pathway was identified in both the hypothalamus and pituitary in comparative transcriptomic studies of the mammalian and poultry HPG axis (53). Proteomic analysis of the hypothalamus and pituitary revealed that Gamma–aminobutyric acid receptor (GABR) related genes were enriched in the Neuroactive ligand–receptor interaction pathway, including GABRA2 in the pituitary, GABRA1 and GABRB2 in the hypothalamus. Gamma–aminobutyric acid (GABA) is a major neurotransmitter in the central nervous system, and it acts mainly through three receptors, GABA_A_, GABA_B_ and GABA_C_. GABA_A_ is a member of the ligand–gated Cl– channel family, while the GABA_B_ receptor is coupled to G proteins (57). The GABRB1 gene identified by the DIABLO method is an important featured variable in the pituitary transcriptome, and it is highly expressed in follicular *BB* ewes than in wild–type, this expressed trend that is consistent with the LHB abundance trend in the proteome. GABA can regulate *BMP2* gene expression through the BMP pathway by activating the GABAB receptor (58). BMP2 is a potential ligand for BMPR1B, and results from *in vitro* experiments in sheep granulosa cells suggest that BMP2 affects estrogen secretion (48). It has been shown that GABA stimulates LH secretion from pituitary cells (59). The knockout of GABA receptors in the hypothalamus may influence the negative feedback regulation of estrogen on the gonadotropin-releasing hormone (GnRH) in mice, consequently impacting the secretion of luteinizing hormone (LH) (60). In summary, the pituitary gland functions as a central component in the HPG axis, integrating signals from the GABA system to regulate ovulation through the modulation of LH or GnRH/LH secretion and interactions with the BMP pathway.

## 5 Conclusions

The investigation of protein abundance in the pituitary of sheep with varying *FecB* genotypes at distinct estrus stages revealed the existence of anterior pituitary hormones (FSH, LH, PRL, and TSH). Mutations in *FecB* have the potential to impact follicular development through the regulation of FSH and LH, consequently leading to alterations in the number of ovulations. CACNA1C and CaMKI are significant marker genes in the pituitary gland that play a crucial role in the regulation of LH and FSH secretion through GnRH. The pituitary integrates signals from the GABA system to modulate ovulation by influencing LH or GnRH/LH secretion and interacting BMP pathway. The integrated analysis of the pituitary gland has yielded novel insights into the significant endocrine regulatory function performed by the pituitary gland in the physiological process of *FecB* mutation impacting ovulation.

## Data availability statement

The datasets presented in this study can be found in online repositories. The names of the repository/repositories and accession number(s) can be found below: National Center for Biotechnology Information Sequence Read Archive (https://www.ncbi.nlm.nih.gov/sra/ Accession no. PRJNA782215) and the OMIX, China National Center for Bioinformation / Beijing Institute of Genomics, Chinese Academy of Sciences (61, 62) (https://ngdc.cncb.ac.cn/omix/ Accession no. OMIX006109).

## Ethics statement

The animal study was approved by the Animal Ethics Committee of the Institute of Animal Sciences, Chinese Academy of Agriculture Science (No. IAS 2019–49). The study was conducted in accordance with the local legislation and institutional requirements.

## Author contributions

XW: Validation, Software, Methodology, Investigation, Formal analysis, Conceptualization, Writing – review & editing, Writing – original draft. XG: Validation, Methodology, Investigation, Funding acquisition, Writing – review & editing. XH: Methodology, Investigation, Writing – review & editing. RD: Project administration, Investigation, Writing – review & editing. XZ: Resources, Investigation, Writing – review & editing. JZ: Resources, Investigation, Writing – review & editing. MC: Supervision, Resources, Project administration, Funding acquisition, Conceptualization, Writing – review & editing.

## Supplementary material

Supplementary Figure 1 Distributions of molecular weight, Protein sequence coverage, and number of unique peptides of proteins identified in the pituitary. **(A)** Distribution of protein molecular weights (kDa). **(B)** Distribution of protein sequence coverage (%). **(C)** Venn diagram displaying the overlap of protein identification by proteomic TMT 6-plex experiments.

Supplementary Figure 2 Illustration of N-integration supervised analysis with DIABLO. **(A)** Sample plot per data set highlighting the integrated protein and mRNA targets. **(B)** Diagnostic scatterplot displaying components 1–2 of each dataset (protein and mRNA), **(C)** Classification performance per component (overall and Balanced Error Rate) for prediction distances (“centroids.dist”, “mahalanobis.dist” and “max.dist”) using repeated stratified cross-validation (3 x 5 fold).

Supplementary Figure 3 The Pearson’s correlation **(A)** and Principal component analysis **(B)** of all samples.

Supplementary Figure 4 Mean ± SEM of serum P4 and E2, and concentration at vaginal sponge removal (0 h) and at the time of euthanasia (45 h and 216 h) in different FecB genotype ewes. Values with different lowercase letters indicate significant differences between genotypes at the same time. (P ≤ 0.05). **(A)** Mean ± SEM of serum P4 (pg/mL), and **(B)** Mean ± SEM of serum E2 (pg/mL).
