# Supplementary figures and images for: Integration analysis of pituitary proteome and transcriptome reveals fertility–related biomarkers in FecB mutant Small Tail Han sheep
Source: Front Endocrinol (Lausanne). 2024 Jul 23;15:1417530. doi: 10.3389/fendo.2024.1417530 (PMC11301337; doi:10.3389/fendo.2024.1417530)

a

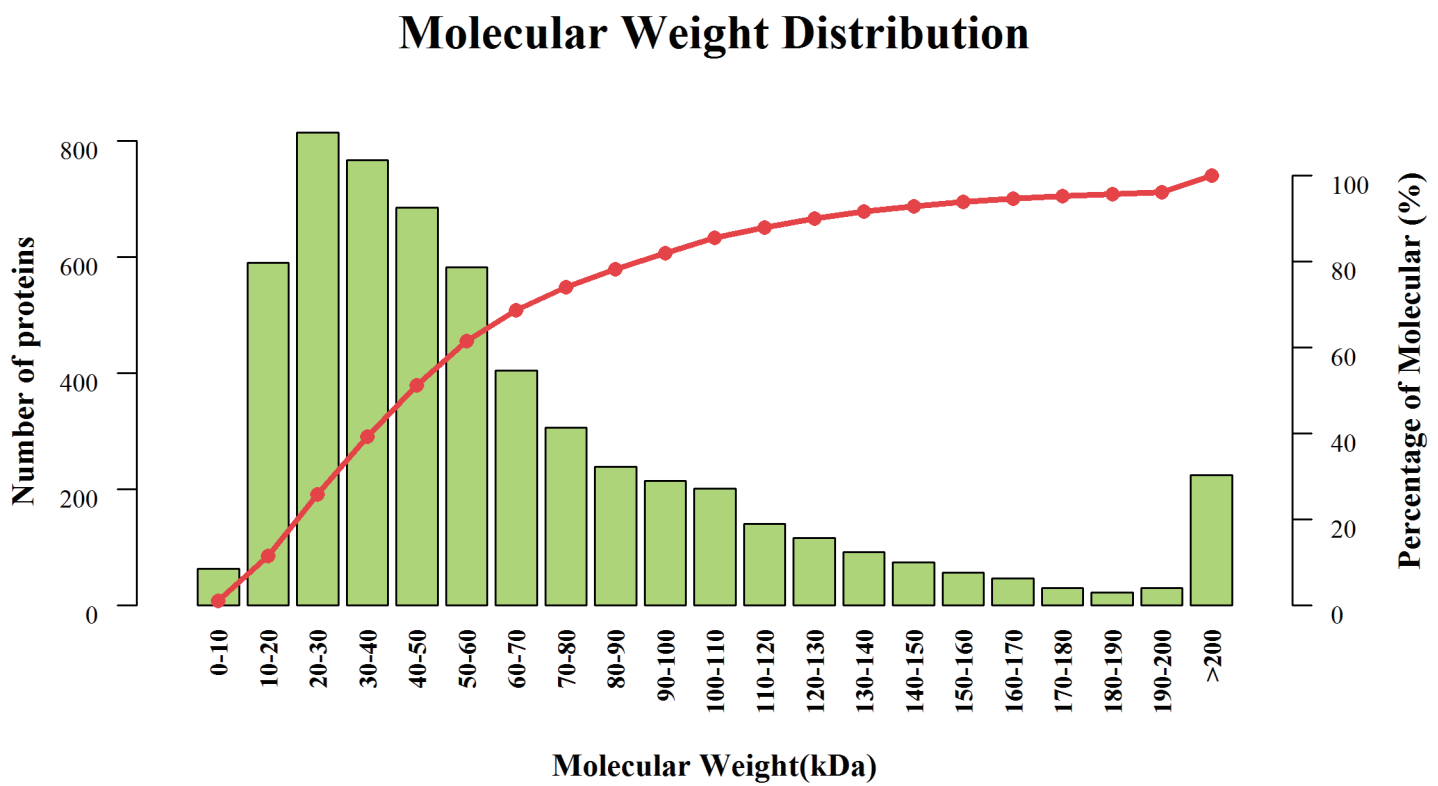

b

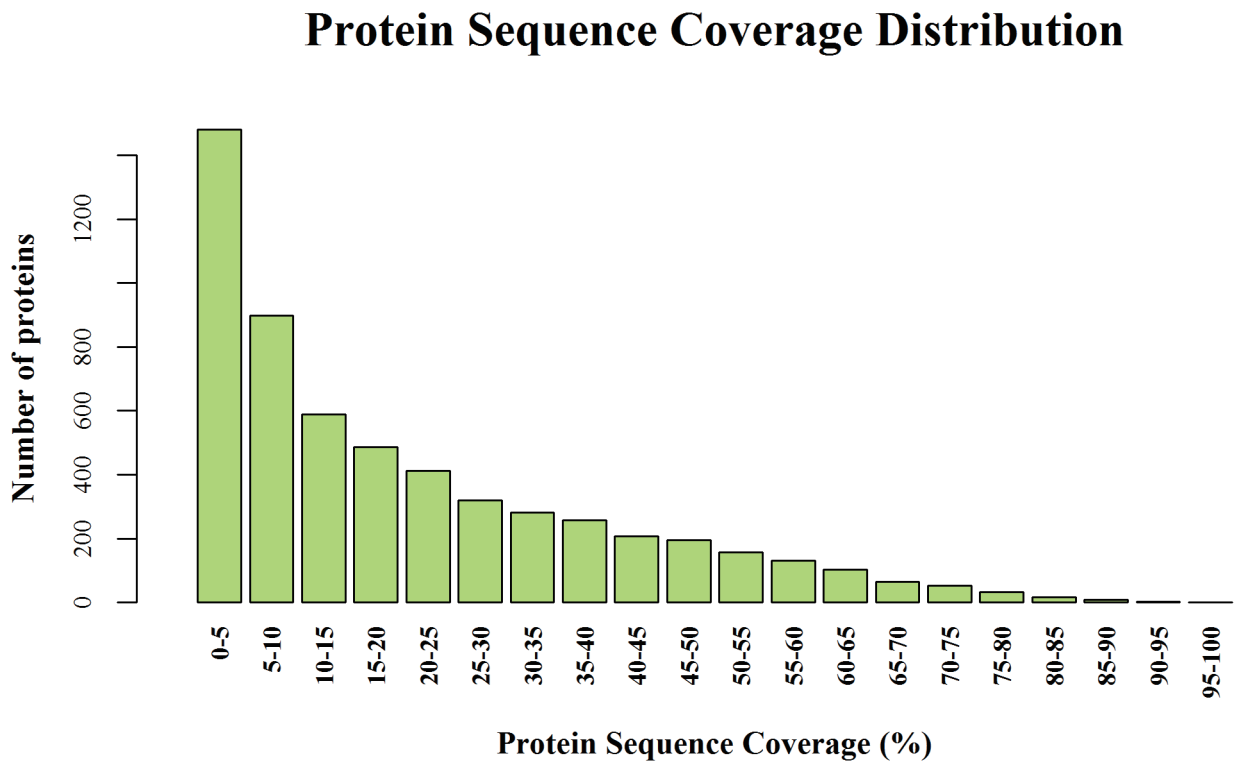

c

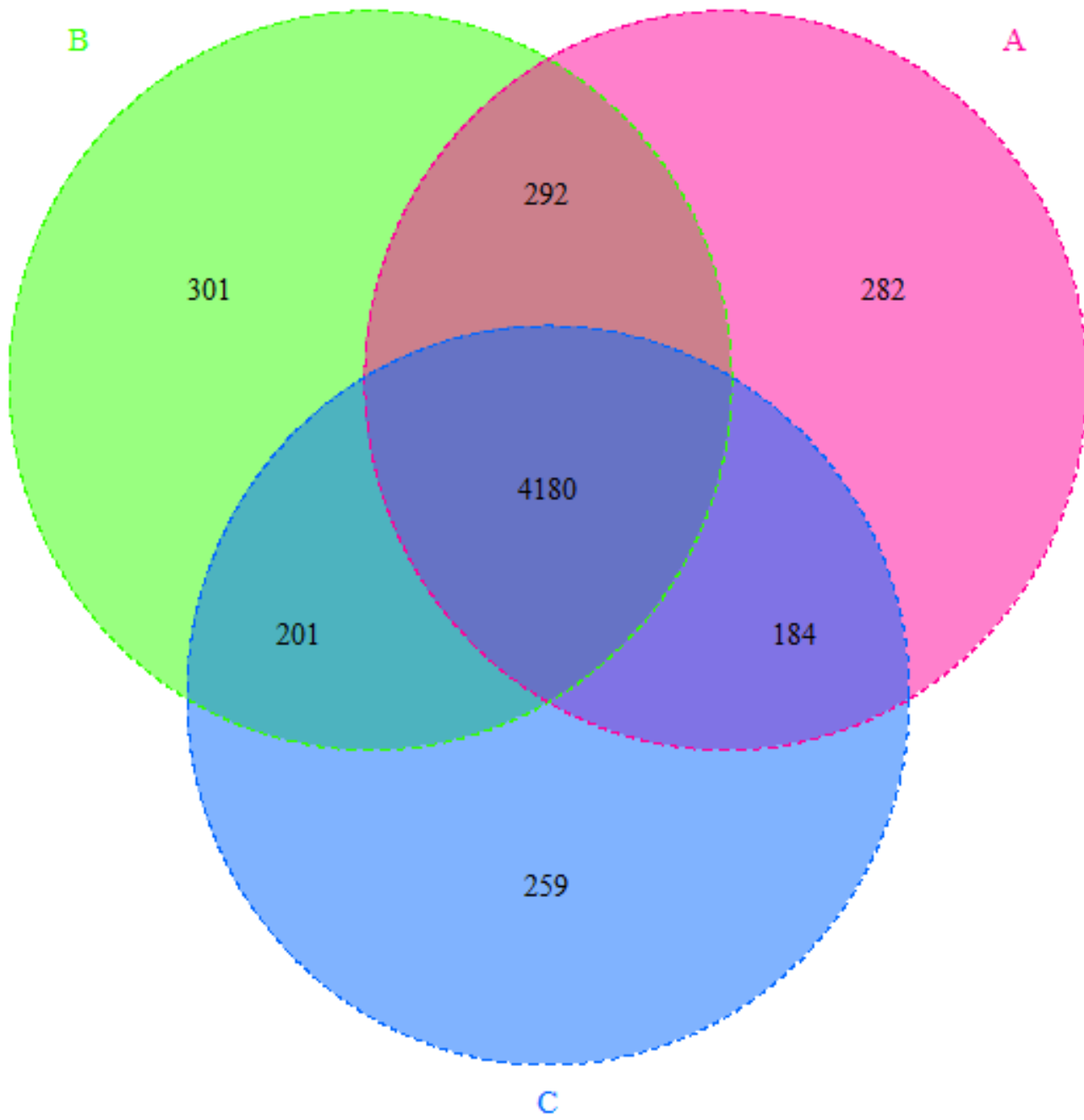

Supplement: Supplementary Figure 1 — Distributions of molecular weight, Protein sequence coverage, and number of unique peptides of proteins identified in the pituitary. (A) Distribution of protein molecular weights (kDa). (B) Distribution of protein sequence coverage (%). (C) Venn diagram displaying the overlap of protein identification by proteomic TMT 6-plex experiments. [file Image_1.pdf]

(a)

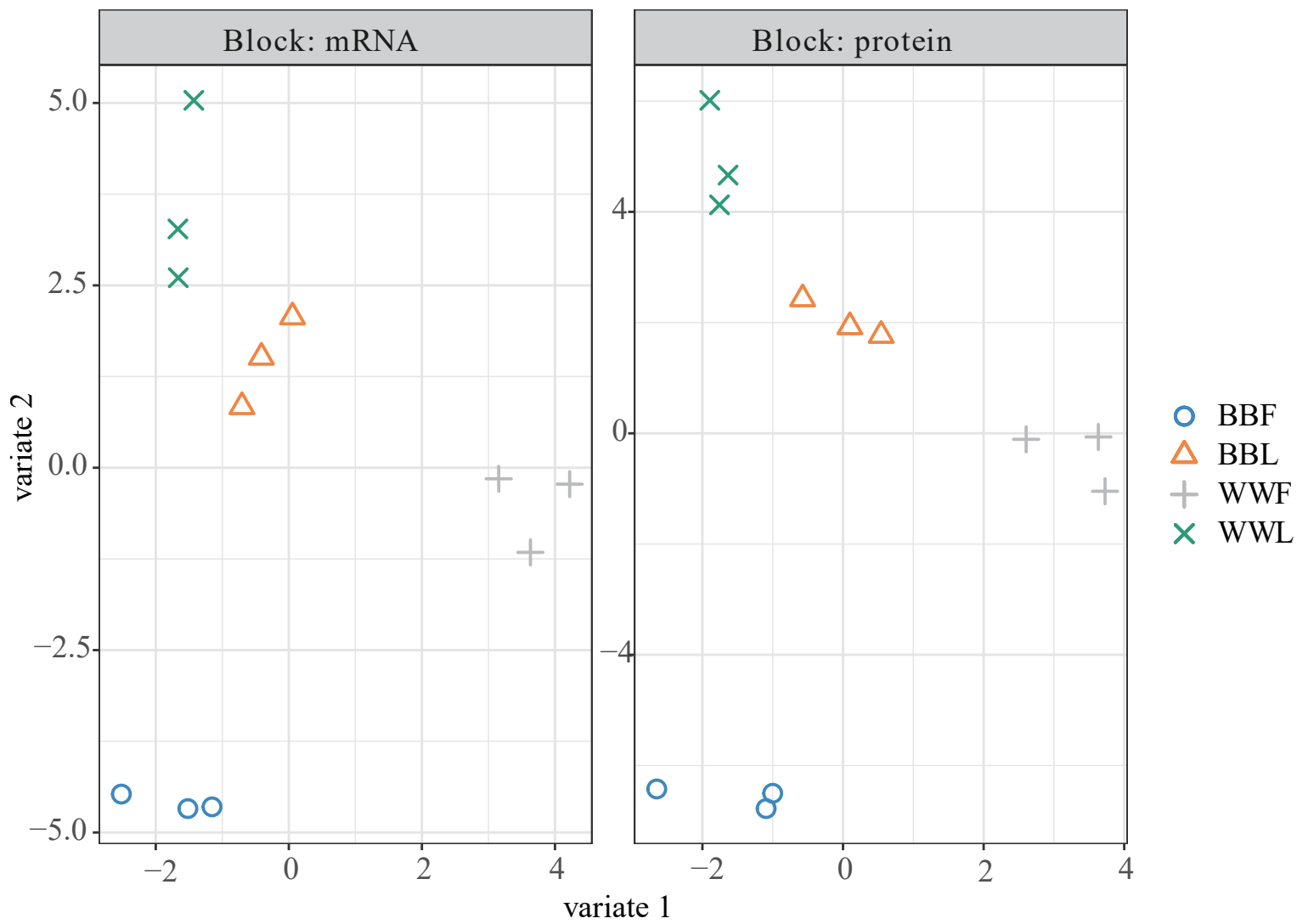

(b)

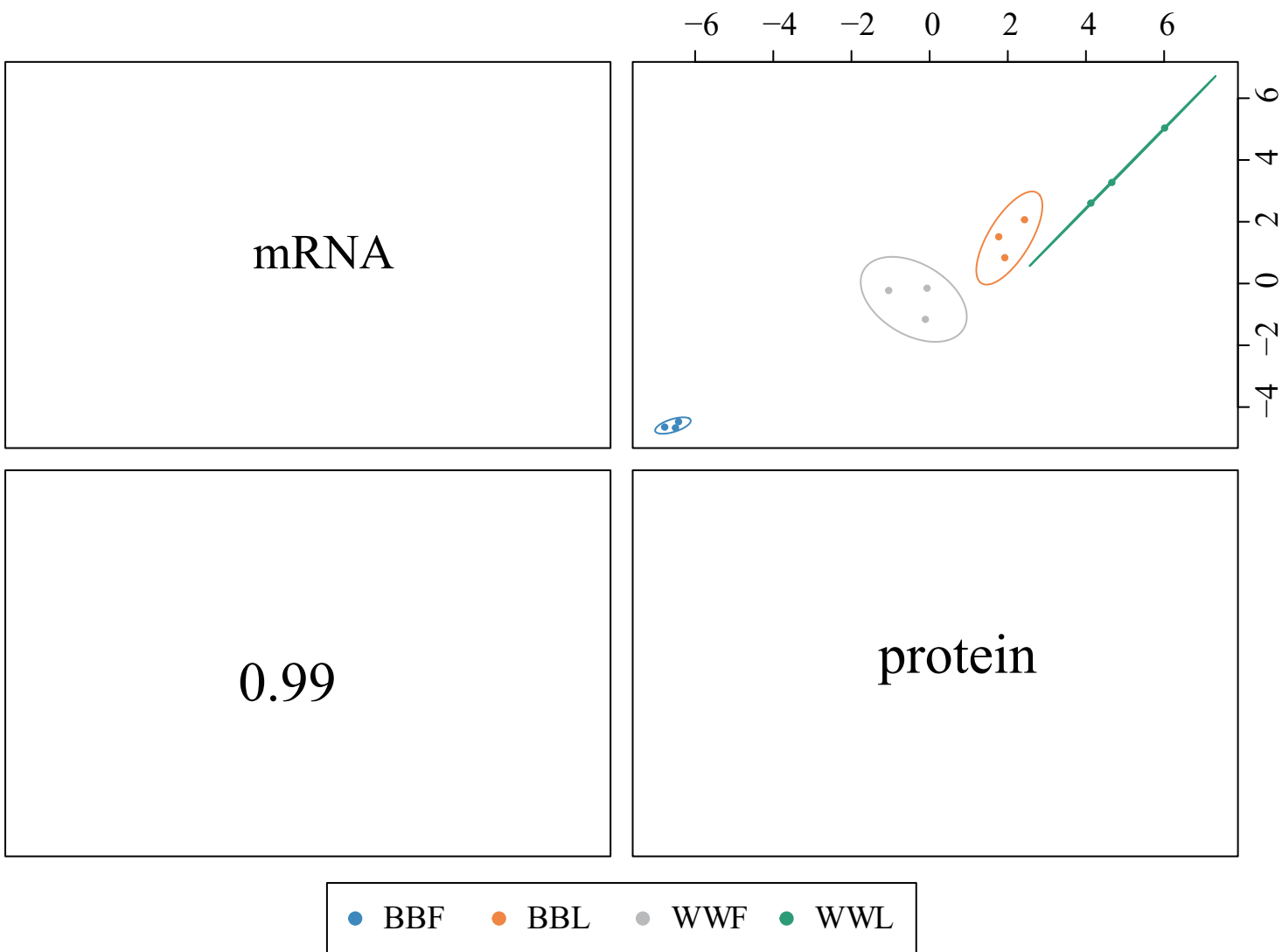

(c)

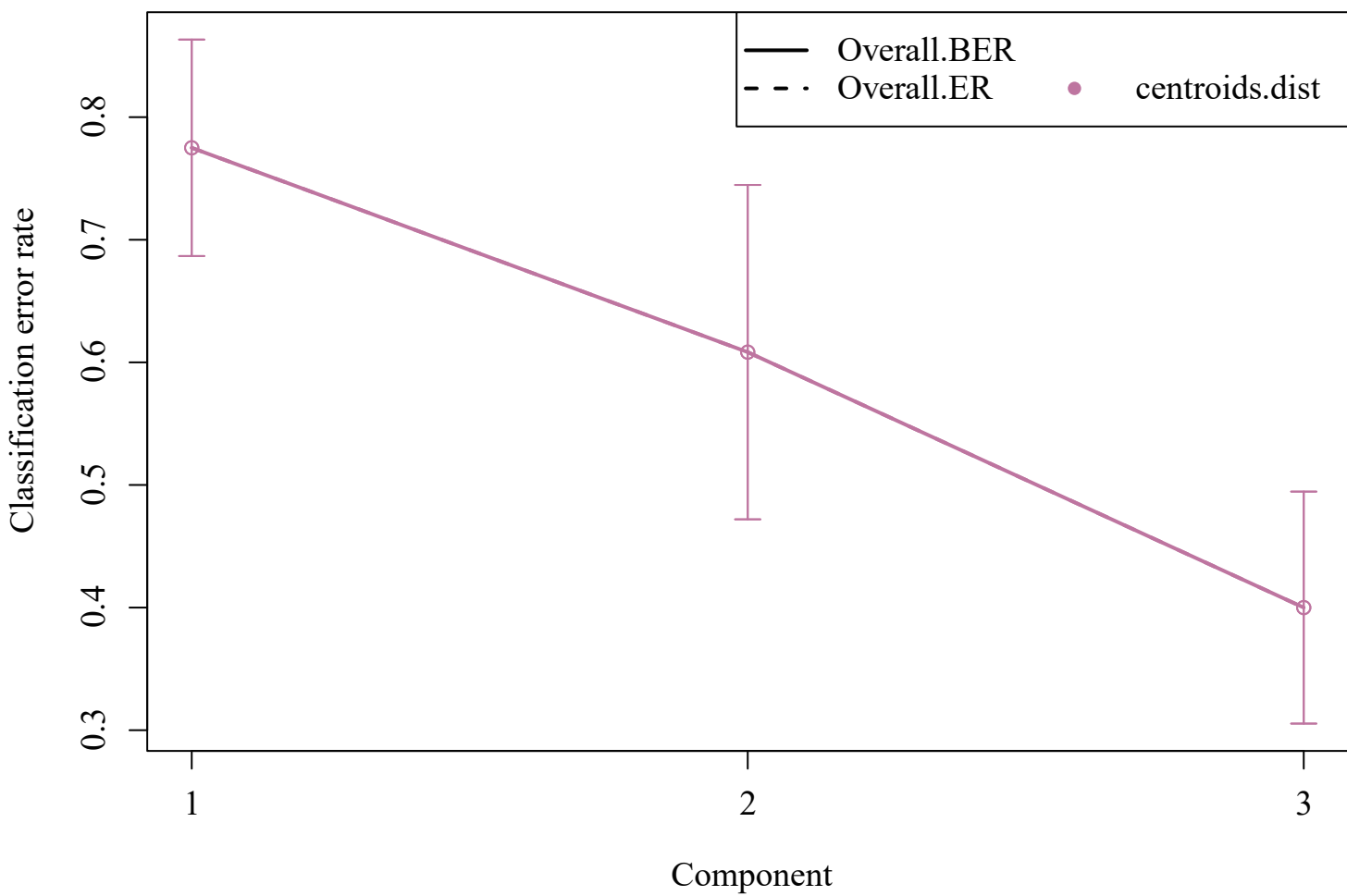

Supplement: Supplementary Figure 2 — Illustration of N-integration supervised analysis with DIABLO. (A) Sample plot per data set highlighting the integrated protein and mRNA targets. (B) Diagnostic scatterplot displaying components 1–2 of each dataset (protein and mRNA), (C) Classification performance per component (overall and Balanced Error Rate) for prediction distances (“centroids.dist”, “mahalanobis.dist” and “max.dist”) using repeated stratified cross-validation (3 x 5 fold). [file Image_2.pdf]

A

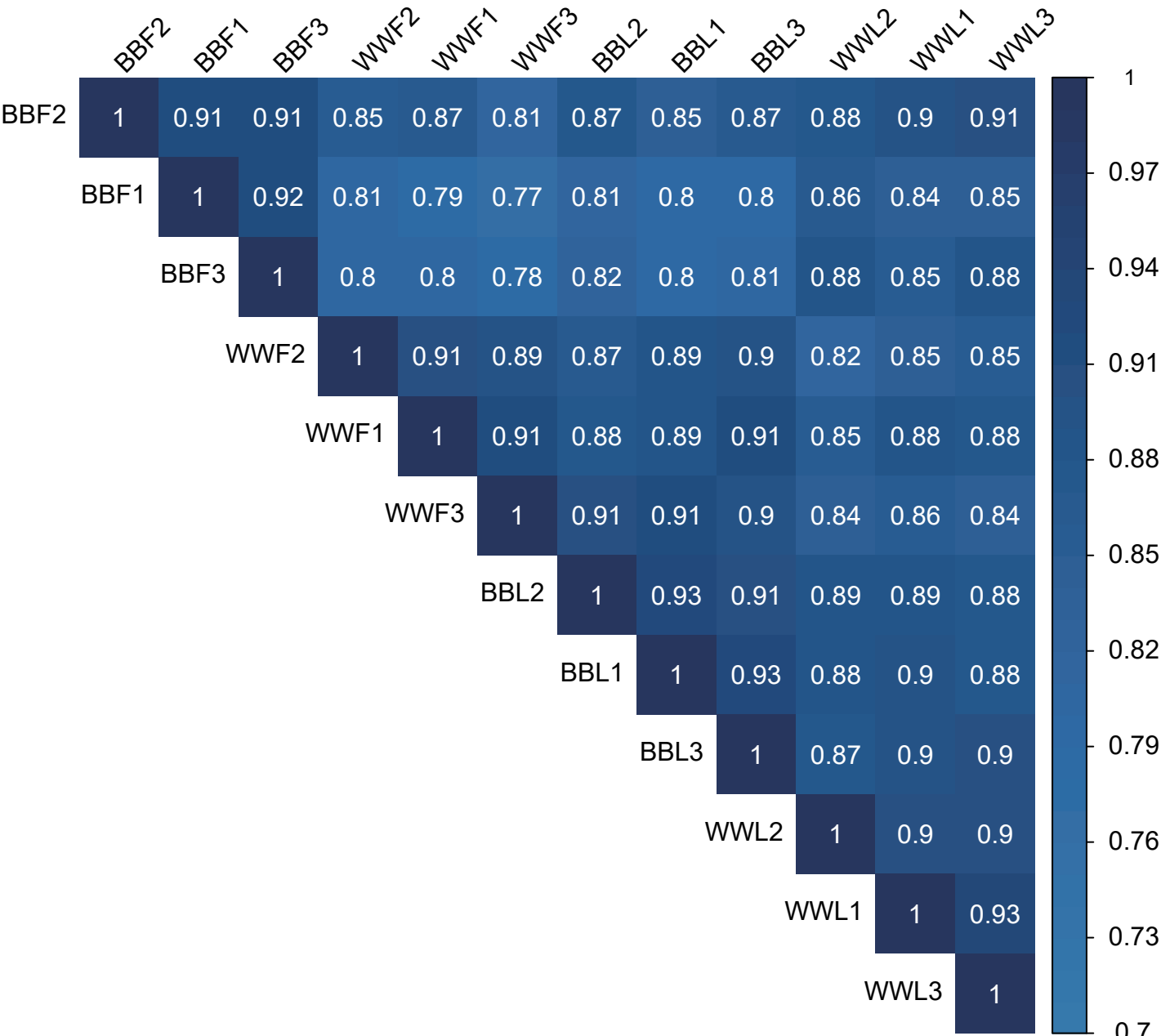

B

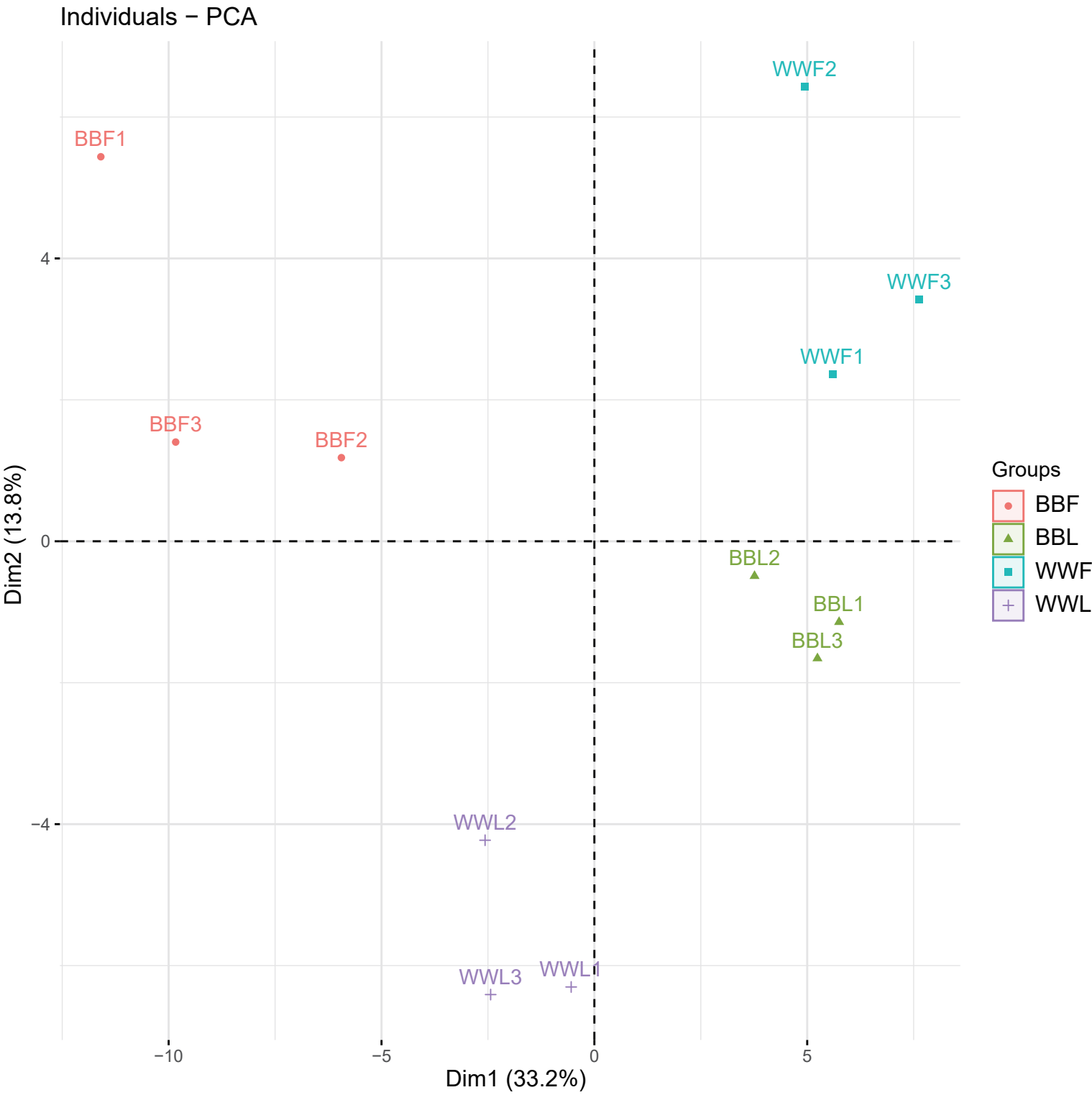

Supplement: Supplementary Figure 3 — The Pearson’s correlation (A) and Principal component analysis (B) of all samples. [file Image_3.pdf]

A

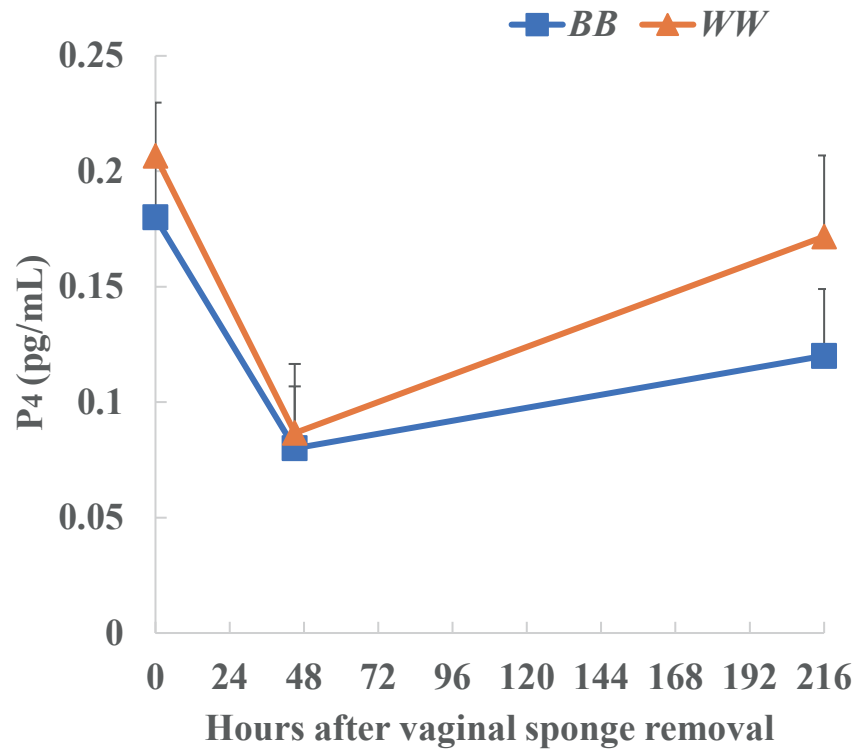

B

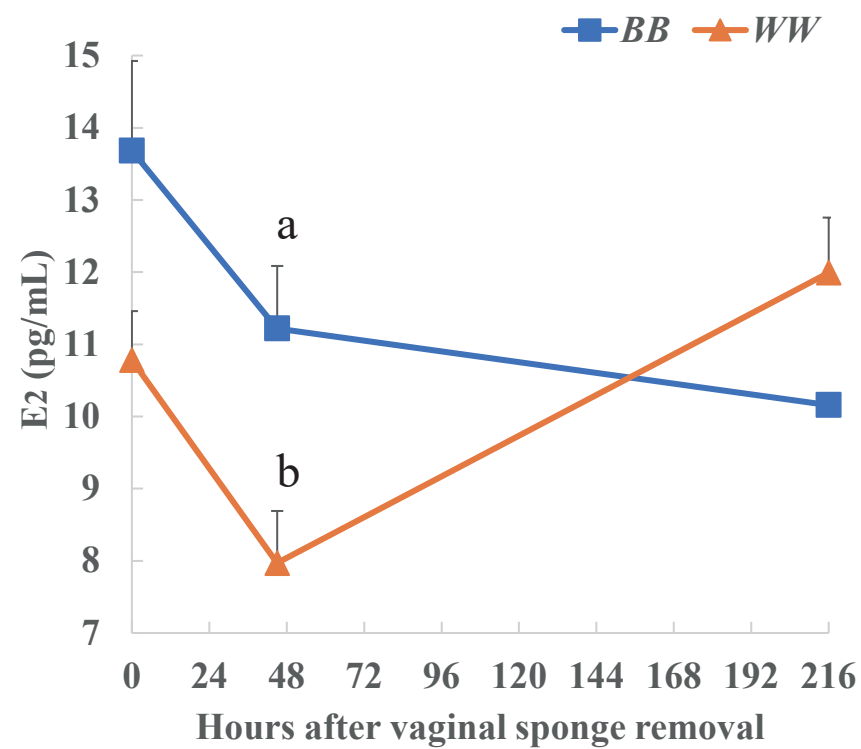

Supplement: Supplementary Figure 4 — Mean ± SEM of serum P4 and E2, and concentration at vaginal sponge removal (0 h) and at the time of euthanasia (45 h and 216 h) in different FecB genotype ewes. Values with different lowercase letters indicate significant differences between genotypes at the same time. (P ≤ 0.05). (A) Mean ± SEM of serum P4 (pg/mL), and (B) Mean ± SEM of serum E2 (pg/mL). [file Image_4.pdf]
